# Too big to purge: persistence of deleterious Mutations in Island populations of the European Barn Owl (*Tyto alba*)

**DOI:** 10.1038/s41437-024-00728-8

**Published:** 2024-10-13

**Authors:** Eléonore Lavanchy, Tristan Cumer, Alexandros Topaloudis, Anne-Lyse Ducrest, Céline Simon, Alexandre Roulin, Jérôme Goudet

**Affiliations:** 1 Department of Ecology and Evolution, University of Lausanne, Lausanne, Switzerland; 2 Swiss Institute of Bioinformatics, University of Lausanne, Lausanne, Switzerland

**Keywords:** Population genetics, Inbreeding, Genomics, Population genetics, Consanguinity

## Abstract

A key aspect of assessing the risk of extinction/extirpation for a particular wild species or population is the status of inbreeding, but the origin of inbreeding and the current mutational load are also two crucial factors to consider when determining survival probability of a population. In this study, we used samples from 502 barn owls from continental and island populations across Europe, with the aim of quantifying and comparing the level of inbreeding between populations with differing demographic histories. In addition to comparing inbreeding status, we determined whether inbreeding is due to non-random mating or high co-ancestry within the population. We show that islands have higher levels of inbreeding than continental populations, and that this is mainly due to small effective population sizes rather than recent consanguineous mating. We assess the probability that a region is autozygous along the genome and show that this probability decreased as the number of genes present in that region increased. Finally, we looked for evidence of reduced selection efficiency and purging in island populations. Among island populations, we found an increase in numbers of both neutral and deleterious minor alleles, possibly as a result of drift and decreased selection efficiency but we found no evidence of purging.

## Introduction

Genomic mutations are the ultimate source of variability in nature, serving as the foundation for evolution and natural selection. However, only a handful of mutations are beneficial for the fitness of an organism, in actuality the majority of mutations are neutral or deleterious (Kibota and Lynch 1996; Kassen and Bataillon 2006; Eyre-Walker and Keightley 2007; Huber et al. 2017). Despite the effects of natural selection, deleterious mutations may still persist within populations. There are two main reasons for this persistence: firstly, through the stochastic segregation of alleles (i.e. drift), which can result in partially deleterious mutations being transmitted by chance and persisting through generations, and secondly the recessive nature of many deleterious mutations can render them invisible to selection when present in a single copy (Charlesworth and Willis 2009; Hedrick and Garcia-Dorado 2016). In natural populations, the persistence and expression of such deleterious effects can be highly detrimental, although the contribution of each of these processes is still mostly uncharacterised in the wild.

Inbreeding is defined as mating between related individuals and can occur from mating among closely related individuals (recent coalescence events), as well as from more ancient coalescence events (even if the current population is mating randomly (Keller and Waller 2002)), such as a historical population bottleneck (Eldridge et al. 1999; Keller and Waller 2002; Furlan et al. 2012; Duntsch et al. 2021), which reduces the effective population size (Ceballos et al. 2018). As a result of inbreeding, individual homozygosity increases, and deleterious recessive effects are expressed, which can eventually result in reduced fitness - referred to as inbreeding depression (Charlesworth and Willis 2009; McQuillan et al. 2012; Pryce et al. 2014; Huisman et al. 2016; Martikainen et al. 2020; Ceballos et al. 2020). While inbreeding depression negatively impacts population fitness, it can paradoxically serve as a mechanism for removing deleterious mutations from the population via purging. As the effect of deleterious recessive alleles are expressed in the homozygous state, selection is more efficient in eliminating them from the population (Xue et al. 2015; Hedrick and Garcia-Dorado 2016; Robinson et al. 2018; Grossen et al. 2020). Hence, the effectiveness of purging is determined by the dominance and deleterious effect associated with each allele where highly deleterious and recessive alleles are more easily purged (Glémin 2003). On the other hand, the purging of mildly deleterious alleles is more challenging, even with long term inbreeding (Wang et al. 1999; Kirkpatrick and Jarne 2000; Day et al. 2003; Glémin 2003; García-Dorado 2012; Hedrick and Garcia-Dorado 2016). Indeed, even though a reduction in population size can lead to purging, it will also increase the effect of genetic drift (Crow 1970; Falconer 1996, 1996; Kardos et al. 2021; Dussex et al. 2023). Consequently, an increase in inbreeding can result in different distributions of deleterious alleles with a decreased frequency of highly deleterious alleles and an increase of mildly and lowly deleterious alleles (Kirkpatrick and Jarne 2000; Glémin 2003; García-Dorado 2012; Willi et al. 2018; Dussex et al. 2023).

Related individuals inherit portions of their genomes that are identical-by-descent (IBD)—inherited by the same common ancestor—which decrease with coalescence time (Thompson 2013; Speed and Balding 2015). There is considerable interest in studying the IBD segments shared between individuals for various purposes (Browning and Browning 2011, 2012), such as inferring demography and population structure (Ralph and Coop 2013; Homburger et al. 2015), as well as identifying deleterious alleles with causal implications (Nakatsuka et al. 2017). It should be noted, however, that the accurate determination of IBD segments between different individuals typically requires phased genetic data, a computationally and statistically challenging task, particularly for non-model organisms. In contrast, detecting IBD segments within individuals is a much simpler task that does not require phased data (since we are examining homozygous segments within an individual, we do not need to know which copy of the chromosome each allele came from). McQuillan et al. (2008) proposed looking for long stretches of homozygosity in polymorphic regions of the genome as proxy for IBD segments within individuals. While short homozygous segments are likely to be homozygous by chance, the probability that long such segments are identical purely by chance is low, thereby increasing the likelihood that they are IBD. McQuillan et al. (2008) introduced the term “Runs of Homozygosity” (ROHs) to describe these long homozygous stretches and showed that they are an informative proxy for an individual’s inbreeding status. The distribution and length of these ROHs can also provide insight into the historical demographic processes of a population (Ceballos et al. 2018) and help disentangle the temporal origin of inbreeding. Indeed, the length of an IBD segment is linked to the time of coalescence (Thompson 2013; Speed and Balding 2015): populations where individuals carry long ROHs may indicate recent consanguineous mating, whereas populations with many short ROHs may suggest an older previous bottleneck event (McQuillan et al. 2008; Ceballos et al. 2018). In wild populations, a number of recent studies have utilised ROHs for inbreeding characterization (Grossen et al. 2020; Stoffel et al. 2021; Humble et al. 2023), inbreeding depression estimation (Stoffel et al. 2021; Duntsch et al. 2021; Kardos et al. 2023; Hewett et al. 2024), demographic history inference (Nigenda-Morales et al. 2023) and homozygosity mapping (Stoffel et al. 2021).

A common method for identifying ROHs involves a windows-based approach, in which consecutive homozygous regions are identified. This method is implemented in PLINK (Purcell et al. 2007; Chang et al. 2015). To improve IBD segment identification accuracy, model-based approaches employing hidden Markov models (HMMs) were developed, such as BCFTools (Narasimhan et al. 2016) and RZooRoH (Bertrand et al. 2019). IBD segments identified with model-based approaches will be hereafter called Homozygous-by-Descent (HBD) segments (Druet and Gautier 2017, 2022; Bertrand et al. 2019).

The barn owl (*Tyto alba*) is a widely distributed nocturnal raptor found throughout Africa and Europe both on the mainland and on islands. Europe’s ecological history has been marked by significant climatic fluctuations, most notably during the last glacial maximum (LGM) approximately 20,000 years ago (Hewitt 1999; Ficetola et al. 2017). As a result, many species, including barn owls, were forced to migrate to warmer southern regions during this period (Hewitt 1999, 2011). Previous research has identified three refugia in southern Europe: (i) the Iberian Peninsula, (ii) the Levant and Anatolia and finally (iii) Italy and Greece (Antoniazza et al. 2010; Burri et al. 2016; Cumer et al. 2021) from which Europe was recolonized when the climate warmed again (Cumer et al. 2021; Machado, Cumer, et al. 2022). As with many recolonization events in other species, the recolonization of Europe by the barn owl likely occurred with bottlenecks at the front of the colonization side, followed by population expansion (Ursenbacher et al. 2015; McDevitt et al. 2022). Such scenarios have been shown to lead to an increased number in deleterious mutations per individual and a reduction in selection efficiency due to the smaller effective population size both with simulated data (Peischl et al. 2013) and with empirical data such as out-of-Africa human expansion (Henn et al. 2016; McCoy and Akey 2016) and postglacial recolonization by salmons (Rougemont et al. 2023). However, since the barn owl populations at the expansion front are geographically close and maintain strong connectivity with the refugial populations (Cumer et al. 2021), the enrichment in deleterious mutations is likely not as strong as what we find in species with more limited dispersal capabilities such as the human out-of-Africa colonization of Europe.

Water bodies can act as barriers to barn owl dispersal and gene flow (Burri et al. 2016; Cumer et al. 2021; Machado, Cumer, et al. 2022; Machado, Topaloudis, et al. 2022). Consequently, island populations exhibit increased isolation (Machado, Cumer, et al. 2022; Machado, Topaloudis, et al. 2022; Cumer et al. 2022) along with higher levels of inbreeding (Machado, Topaloudis, et al. 2022; Cumer et al. 2022) compared to continental populations. This reduced gene flow in conjunction with a smaller population size can reduce the effectiveness of natural selection. Because of its unique demography, which includes a variety of population sizes, and well-studied recolonization history, the barn owl is well suited for studying the effect of effective population size, inbreeding, and purging in the wild.

In this study, we analyze 502 high-quality sequenced genomes of barn owls from 19 different populations throughout Europe. We characterize the inbreeding status and landscape of homozygosity-by-descent (HBD) segments by using HBD segment-based inbreeding coefficients and distributions. Island populations and, to a lesser extent, populations outside of refugia have experienced founder effects through colonization. As a result, we expect their effective population sizes to be smaller, resulting in a reduction in selection efficiency. Hence, we hypothesize that deleterious mutations are more prevalent within islands and in recolonized populations. In addition, we look for evidence of purging of highly deleterious alleles in these same populations.

## Materials and methods

All code used in this study can be found on the following GitHub repository: https://github.com/EluLava/InbreedingTytoalbaEurope2023.

### Sampling, sequencing, and genotyping

**Fig. 1:**
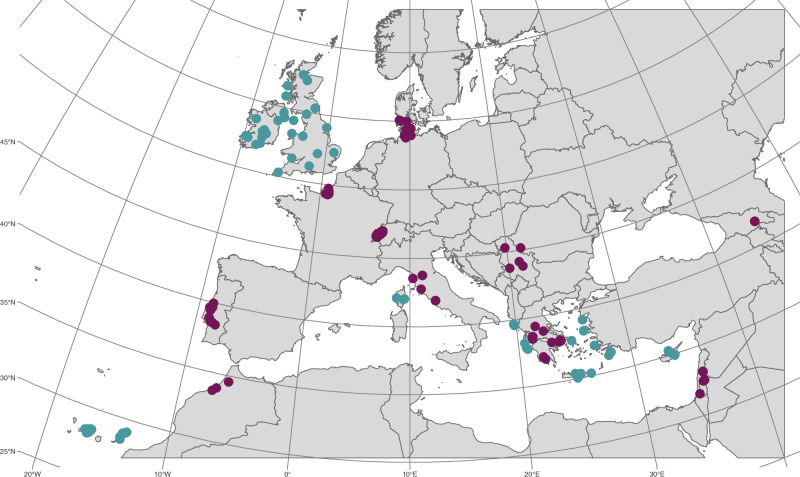
Map of samples’ locations. Continental samples are shown in purple and island samples in blue.

A total of 502 barn owls (*Tyto alba*) were sampled from 19 populations (details on sample sex and location in Table S1, Fig. 1 and original samples description in Cumer et al. (2021); Machado, Cumer, et al. (2022); Machado, Topaloudis, et al. (2022); Cumer et al. (2022); Topaloudis et al. (2024) and Cumer et al. (2024): 346 individuals from Switzerland (CH), 15 from Grand-Britain (GB), 12 from Ireland (IR), 11 from continental Greece (GR) and the Aegean islands (AE) each, 10 from Denmark (DK), Israel (IS), Portugal (PT), Italy (IT), Crete (CT), Cyprus (CY), East canary (EC) and West canary (WC) each, 6 from Georgia (GE), 5 from France (FR), Serbia (SB) and the Ionian islands (IO) each, 3 from Morocco (MA) and Corsica (CO) each. Figure 1 shows the 502 sample locations; samples from continental populations are shown in purple and individuals from island populations in blue. We further categorised the continental populations as either: refugium populations, defined as those that were present during the last glacial maxima or recolonized populations. Assignments to each category based on previous studies (Antoniazza et al. 2010; Burri et al. 2016; Cumer et al. 2021; Machado, Cumer, et al. 2022), were as follows refugium populations: PT, MA, IT, GR and IS, recolonized populations: CH, FR, DK and SB. Although the Mediterranean islands were suitable habitats during the last glacial maxima (Machado, Cumer, et al. 2022), we excluded all islands from the comparison for refugia and recolonized populations due to their small size which could bias the results.

These samples were collected for previous projects with an objective of generating high quality genomic data for these individuals, see Cumer et al. (2021); Machado, Cumer, et al. (2022); Machado, Topaloudis, et al. (2022); Cumer et al. (2022); Topaloudis et al. (2024); Cumer et al. (2024) for a detailed description. In brief, DNA was extracted from individual blood and tissue samples with the DNeasy Blood & Tissue kit (Qiagen). Libraries were prepared using TruSeq® DNA PCR-Free Library Prep or Nextera XT DNA Library Preparation Kit (Illumina GMBH, Switzerland). Whole-genome resequencing was then carried out at the Lausanne Genomic Technologies Facility (GTF, University of Lausanne, Switzerland) using Illumina HiSeq 2500 PE (100–150 bp) high-throughput sequencing (mean coverage: 16X). Raw reads from autosomal chromosomes were trimmed and aligned to the *Tyto alba* v.4 reference genome (Machado, Cumer, et al. 2022) using Trimommatic (v0.39) (Bolger et al. 2014) and BWA-MEM (v.0.7.17) (Li and Durbin 2009). ‘Base quality score recalibration’ (BQSR) was performed with high-confidence calls from GATK(v4.2.6.1). Individual genotypes were called with GATK’s *HaplotypeCaller* method and joint-calling was applied on all 502 samples using *GenotypeGVCFs*.

Variants were filtered with GATK as follows: QD < 2.0, FS > 60.0, MQ < 40.0, MQRankSum < −12.5, ReadPosRankSum < −8.0, SOR > 3.0. An additional ‘mappability’ filter was performed to discard any regions for which the mapping quality was low. Individuals with a genotype depth (GD) < 5 or *>* mean individual depth plus three times its standard deviation were removed. Sites with minor allele count (MAC) < 3 and fraction of missing individuals’ genotypes *>* 0.10 were also removed. For the subsequent analysis we kept only bi-allelic SNPs to allow for HBD segment characterization in RZooRoH (Bertrand et al. 2019) resulting in a total of 14,093,173 high-quality bi-allelic SNPs in 502 individuals.

### HBD segments identification

IBD segments were called on autosomes only and using the RZooRoH package (v.0.3.1) (Druet and Gautier 2017, 2022; Bertrand et al. 2019). Such segments are hereafter referred to as HBD segments because they were called with a model-based approach (see introduction). Our model included 13 HBD classes and 1 non-HBD class with rates (R) of 2, 4, 8, 16, 32, 64, 128, 256, 512, 1024, 2048, 4096 and 8192 for the HBD classes and 8192 for the non-HBD class (Druet and Gautier 2017). These HBD classes correspond to different coalescence event ages and the rate corresponds to the expected number of generations (g) since the coalescence event, divided by two. We considered the ‘most probable HBD segments’ estimated by RZooRoH Viterbi algorithm for HBD segment distributions analyses. HBD segment distributions were then obtained by quantifying the mean sum of HBD segment lengths (among individuals) falling into the autozygous HBD classes. We considered a HBD class as autozygous if the rate was less than 1024 (i.e. if the coalescence event occurred during the last 512 g) (Browning and Browning 2012).

In order to take into account the recombination rate along the genome, we used recombination maps built with Lep-MAP3 (Rastas 2017) and described in (Topaloudis et al. 2024). We interpolated the genetic positions of our SNPs between each SNP present in the recombination map via a linear model. If no map was available for a specific super scaffold, we assumed a constant recombination rate of 2 × 10 − 8 (the average recombination rate between adjacent base pairs among the rest of the super scaffolds).

### Estimating inbreeding coefficients

The *F*_*HBD*_ inbreeding coefficient is defined as the average probability (among markers) of belonging to a HBD segment and were obtained with the *cumhbd* function from the RZooRoH package with a T value of 1024. This value means that only HBD segments coalescing less than 512 g ago are considered autozygous (i.e. IBD) (Browning and Browning 2012).

*F*_AS_ is an allele-sharing-based estimator of inbreeding described in (Weir and Goudet 2017; Zhang et al. 2022). It corresponds to the average allele-sharing for an individual (taking value 1 if the individual is homozygous and 0.5 if heterozygous at a specific locus), scaled by the mean allele-sharing between individuals of the population. Consequently, *F*_AS_ was estimated separately in each population. In addition, since the Swiss population (CH) contained related individuals, we only considered individuals with relatedness *<* 0.05 for estimating the mean between individual allele-sharing for this population (the list of unrelated individuals can be extracted from Table S1). Using this unbiased average, we estimated individual inbreeding coefficients for all the 502 individuals.

### Probability of belonging to a HBD segment

We followed the procedure in (Stoffel et al. 2021) to estimate the probability of belonging to a HBD segment along the genome. For this analysis, we excluded any super-scaffold with less than 10,000 SNPs. The probability of belonging to a HBD segment was first estimated for each variant position as the sum of probabilities to belong to any HBD class estimated from the *hbdp* object from the output of the *zoorun* function from the RZooRoH R package. Average probabilities were then estimated via 20Kb overlapping sliding windows of 100Kb with the windowscanr R package.

### Nucleotide diversity and effective population size

The nucleotide diversity *π* was estimated with the *pi.dosage* function from the hierfstat R package (Goudet 2005). The effective population size (*N*_e_) was then estimated as *π* divided by four times the mutation rate (*µ* = 4.6 × 10 − 9 (Smeds et al. 2016)). To obtain confidence intervals, we divided the genome into 1 Mb segments and performed 1000 bootstraps for each population separately.

### Variant annotation

Variant annotation was performed with SnpEff (Cingolani et al. 2012) on the filtered complete dataset (14,093,173 bi-allelic SNPs). We used the *build* method of SnpEff on the NCBI latest version of the *Tyto alba* assembly, as at the time of publication none was available. Variant annotation was then performed with the *eff* method from SnpEff after filtering sites with missing data greater than 10%. Bi-allelic variants were classified into four categories using SnpEff: neutral, lowly deleterious, mildly deleterious, and highly deleterious. The neutral variants consist of changes to non-coding regions (including pseudo-genes), UTR regions and regions where it is difficult to predict the impact of the variant. Lowly deleterious variants refer to mutations that are harmless or unlikely to alter protein behavior, including synonymous mutations, nonsynonymous variants that change one or more amino acids but have similar properties to the originals, or changes in start (or stop) codons into different start (or stop) codon types. Mildly deleterious variants included mutations that may affect protein effectiveness, such as changes in the amino acid sequence leading to altered protein properties. Finally, highly deleterious mutations concerned variants that have a large (disruptive) impact on the protein, most likely resulting in truncation or loss of function. The highly deleterious variants included, for example, loci implicated in protein-protein interactions (i.e. amino acids which are in contact within the same protein, possibly involved in structural conformation), rare amino acids that are likely to result in protein loss of function, variants mutating stop (or start) codons into non-stop (or non-start) codons (and vice versa). A more detailed description of these four categories can be found in the SnpEff documentation (Cingolani et al. 2012).

### Accumulation of minor alleles

Because populations with small effective sizes are more sensitive to the effects of drift, we expect the island populations to be enriched in fixed minorly deleterious alleles. To assess this assumption in our populations, we used the *R*_*XY*_ and *R*^2^_*XY*_ statistics described in (Do et al. 2015). These statistics aim at detecting an asymmetry in the number of minor alleles between two groups of genomes (*X* and *Y*) and count how many of these alleles (*L*_*XnotY*_) or expected homozygous alleles (*L*^2^_*XnotY*_) are present in one group of genomes, but not the other and are formally defined as follows:

1 $${L}_{{XnotY}}=\mathop{\sum}\limits_{i}\left({d}_{X}^{i}/{n}_{X}^{i}\right)\left(1-{d}_{Y}^{i}/{n}_{Y}^{i}\right)$$

2 $${L}_{{XnotY}}^{2}=\mathop{\sum}\limits_{i}\frac{2{d}_{X}^{i}\left({n}_{X}^{i}-{d}_{X}^{i}\right)}{{n}_{X}^{i}\left({n}_{X}^{i}-{d}_{X}^{i}\right)}\left(1-\frac{2{d}_{Y}^{i}\left({n}_{Y}^{i}-{d}_{Y}^{i}\right)}{{n}_{Y}^{i}\left({n}_{Y}^{i}-{d}_{Y}^{i}\right)}\right)$$

with ${d}_{X}^{i}$ and ${n}_{X}^{i}$ being respectively the number of (global) minor alleles and the total number of (haploid) genomes at site *i* in population *X* and ${d}_{Y}^{i}$ and ${n}_{Y}^{i}$ the number of minor alleles and the total number of (haploid) genomes at site *i* in population *Y*. The ratios between the two groups of populations are then calculated as:

3 $${R}_{{XY}}=\frac{{L}_{{XnotY}}}{{L}_{{YnotX}}}$$

4 $${R}_{{XY}}^{2}=\frac{{L}_{{XnotY}}^{2}}{{L}_{{YnotX}}^{2}}$$

We estimated both statistics for variants included in exons only and for each allele category (neutral, highly deleterious, moderately deleterious and lowly deleterious) separately. To account for different demographic histories and structure within each group of populations (Do et al. 2015; Xue et al. 2015; Grossen et al. 2020), we further divided these ratios by the same ratios estimated for intergenic variants only:

5 $${R}_{{XY}}^{{\prime} }=\frac{{R}_{{XY}}^{{class}}}{{R}_{{XY}}^{{inter}}}$$

6 $${R^{\prime} }_{{XY}}^{2}=\frac{{R}_{{XY}}^{{2}^{{class}}}}{{R}_{{XY}}^{{2}^{{inter}}}}$$

Consequently, the ${R}_{{XY}}^{{\prime} }$ and ${R^{\prime} }_{{XY}}^{2}$ statistics here indicate an enrichment (if R < 1) or depletion (if R > 1) of (homozygous) minor alleles in population *Y* compared to population *X* in regards to what was observed for intergenic mutations. If natural selection has been equally efficient in both populations, ${R}_{{XY}}^{{\prime} }$ and ${R^{\prime} }_{{XY}}^{2}$ should be equal to one. Similarly, if selection has been equally efficient for lowly, mildly and highly deleterious alleles, the ${R}_{{XY}}^{{\prime} }$ and ${R^{\prime} }_{{XY}}^{2}$ ratios should be the same for each mutation category.

For this analysis, we compared the enrichment in minor alleles rather than derived alleles because we could not confidently identify the ancestral and derived allele in each locus. In order to avoid any bias due to sampling, minor alleles were estimated globally via 1000 bootstraps by sampling only unrelated individuals. The allele which was identified as the minor allele in the majority of the bootstraps was used as the minor allele for this site. Polarization of a subset of 1,373,932 of the variants has been performed in Machado, Cumer, et al. (2022). We used this subset to confirm whether the minor alleles we identified correspond to the inferred derived alleles. Both methods identified the same allele for 87% of the sites. This fraction linearly decreased with minor allele frequency (being at 95% for sites with MAF < 0.05 and at 0.53 for sites with MAF between 0.45 and 0.5, illustrated in Fig. S1). As the majority of deleterious alleles are likely to be at low frequencies (Pritchard 2001) we assume that the minor allele may be used as a proxy for the derived allele for this analysis.

## Results

### Inbreeding status

**Fig. 2:**
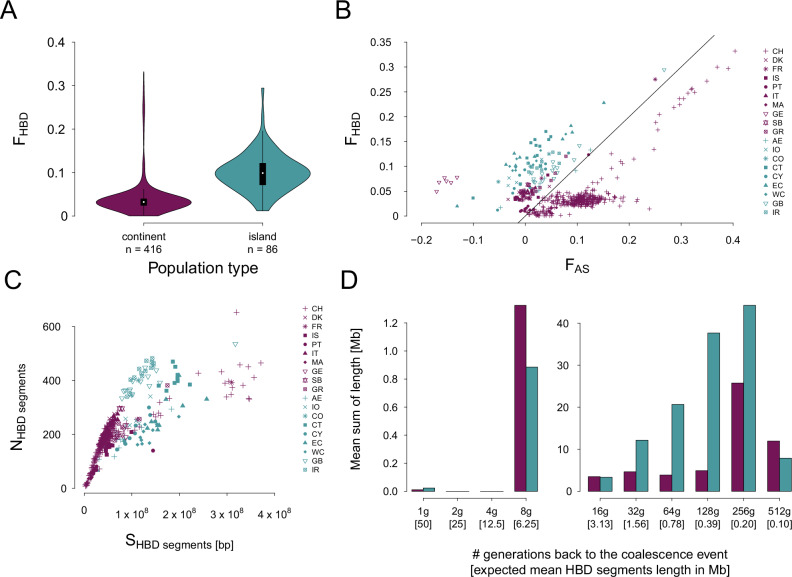
Inbreeding difference between mainland and island populations. For all panels, continental populations are shown in purple and island populations in blue. **A** *F*_*HBD*_ distributions from continental and island populations. *F*_*HBD*_ considers a marker as autozygous if the coalescence event is up to 512 generations ago. **B** scatter plot of *F*_*HBD*_ against *F*_*AS*_. Each point represents one individual and its shape indicates which population it comes from. The black line is the identity line (x = y). **C** number of HBD segments (*N*_*HBD*_) as a function of the mean length of HBD segments (*S*_*HBD*_) in base-pair. Each point represents one individual and its shape indicates which population it comes from. **D** HBD segments distributions from continental populations and island populations. The *y*-axis represents the mean sum of length (among individuals) falling into the different categories of HBD segments (represented in the *x*-axis).

We first quantified individuals’ inbreeding via an inbreeding coefficient (*F*_*HBD*_) based on IBD segments identified with a model-based approach and thus denoted HBD segments. Individual values of *F*_*HBD*_ ranged between 0.001 and 0.346, with a mean of 0.063 (Table S2). The mean *F*_*HBD*_was significantly higher for island populations (mean *F*_*HBD*_ = 0.101) compared to continental populations (mean *F*_*HBD*_ = 0.041, Wilcoxon rank test; W = 3486, *p*-value < 2.2e-16; large effect size: 0.525, Fig. 2A). The differences between continental and island populations begin at coalescence events older than 16 generations (g) ago (corresponding to HBD class 4) and continue to 4096 g (Fig. S2). F_HBD_ distributions per population can be found in Fig. S3. There is little difference between the inbreeding coefficients for populations from continental refugia (mean *F*_*HBD*_ = 0.041; median *F*_*HBD*_ = 0.039) and continental recolonized populations (mean *F*_*HBD*_ = 0.041; median *F*_*HBD*_ = 0.032, Fig. S4A) but their F_HBD_ distributions remain significantly different (W = 10’063, *p*-value = 0.01275; effect size: 0.122, considered small).

To identify the original time of the inbreeding event, we extend the approach proposed by Clark et al. (2019) (more details on the comparison of both methods in supplementary material, section comparing *F*_*AS*_ and *F*_*HBD*_) by contrasting two inbreeding coefficients, one representing the quantity of alleles shared within individuals (*F*_*AS*_) and another the fraction of the genome which is IBD (*F*_*HBD*_). We compared these individual inbreeding coefficients between populations and population types (i.e. continental and island populations), see Fig. 2B. *F*_*AS*_ represents the within individual matching of alleles relative to the mean between individuals in the population. Fundamentally, this quantifies the degree to which alleles are associated at random in individuals, where *F*_*AS*_ > 0 shows that the same variant is associated more often than expected by chance. Individuals below the identity line, i.e. when *F*_*AS*_ is higher than *F*_*HBD*_, are subject to population structure which promotes inbreeding and indicates that the population is not homogeneous. On the contrary, in individuals where *F*_*HBD*_ is higher than *F*_*AS*_ their inbreeding originates in ancient coalescence events (such as ancient population relatedness due to small effective population size) rather than recent mating between relatives. In Fig. 2B, we show that all individuals coming from island populations fall above this identity line with an *F*_*HBD*_ higher than *F*_*AS*_. This indicates that their inbreeding mostly comes from ancient relatedness, likely due to small effective population size as a result of island isolation. In addition, among island populations, individuals from GB are closest to the identity line, reflecting this islands larger size and thus its higher population size. Within island populations, the individuals with the highest *F*_*HBD*_ are from CT, CY and EC. Four island individuals harbor low *F*_*HBD*_ and negative *F*_*AS*_ values indicating a lower matching of alleles compared to the rest of the population (one from each of: EC, CT, CY and AE). Finally, two island individuals (one from EC and one from GB) show very high *F*_HBD_ and *F*_*AS*_ suggesting that they come from mating between closely related individuals.

In continental populations, there is no strong difference between refugium populations and re-colonized populations (except for the Swiss individuals) (Fig. S4B). Most Swiss individuals cluster below the identity line indicating the presence of unaccounted structure. Indeed, individuals in this population tend to be within families, rather than a random subset of individuals. If we control for family structure in the Swiss population by filtering out individuals with allele-sharing relatedness above 0.05, individuals are drawn closer to the line (Fig. S5). Fourteen (out of 346) Swiss individuals have extreme values of both *F*_*AS*_ and *F*_*HBD*_ and fall close to the identity line, indicitive of a recent close inbreeding event. This is concordant with estimates obtained from an observational pedigree in the long time monitored Swiss population (see Supplementary Material and Fig. S6). PT individuals cluster close together and near to the identity line. GE individuals cluster together with *F*_*HBD*_ between 0.05 and 0.1 and *F*_*AS*_ around −0.15. Concerning the remaining continental populations, the three MA samples are below the identity line. We also observe a few highly inbred individuals (one from EC, one from PT and one from FR) towards the top right part of the graph and the remaining individuals have lower values of *F*_*HBD*_ < 0.05 and *F*_*AS*_ $\approx$ 0.

The origin of inbreeding can also be inferred from the distribution of HBD segments distributions. Consequently, as a complementary approach to the above analysis we quantified the mean number of HBD segments (*N*_*HBD*_) according to the mean length of all HBD segments (*S*_*HBD*_) for each individual, Fig. 2C. In general, inbred individuals (individuals with higher *F*_*HBD*_) have more HBD segments but also longer segments. Island individuals have on average slightly more HBD segments compared to continental populations and longer HBD segments (Fig. 2D). Very few long HBD segments (indicative of inbreeding events occurring in the last 8 g) were found in either continental or island populations, suggesting that there is almost no recent inbreeding. However, island populations displayed a higher sum of lengths for medium-sized HBD segments coalescing between 16 g and 128 g. Interestingly, refugium populations were slightly enriched in HBD segments coalescing 64 g and 128 g ago while recolonized populations were enriched in HBD segments coalescing 8 g and 512 g ago (Fig. S4C, D). The increased sum of length of HBD segments coalescing 8 g ago in the continental and refugia populations are driven by the few inbred Swiss individuals. *F*_*HBD*_ and HBD segments distribution estimations per population can be found in supplementary materials (Figs. S3, S7).

### Effective population size

We also estimated the effective population size (*N*_e_) per population as this is an important measure of population diversity (Table 1). The absolute values are unusually large but the relative comparisons should still be valid. In general, continental populations show slightly higher *N*_e_ estimations compared to island populations, with three notable exceptions. The continental GE population, which is composed of related individuals, thus decreasing the estimated *N*_e_; the island AE population, which has very strong gene flow with the GR population; and the island CY population (explored in the discussion). Within island populations, IR, followed by CT displayed the lowest *N*_e_ estimation. On the contrary, the AE and CY populations showed the highest *N*_e_. Concerning continental populations, the refugium populations (PT, MA, IT, GR and IS) displayed the highest *N*_e_ estimation (except IT).

**Table 1:** Effective population sizes (*N*_*e*_).

Population Type	Population	Mean Ne	SE
Continent	PT	118,271	1018
Continent	MA	116,587	978
Island	AE	114,640	975
Continent	IS	114,633	967
Continent	GR	114,362	968
Continent	CH	112,976	974
Continent	DK	112,854	984
Continent	IT	112,016	980
Island	CY	111,752	950
Continent	SB	111,731	973
Continent	FR	111,226	985
Island	WC	108,748	934
Island	IO	108,658	947
Island	GB	107,886	937
Island	EC	107,273	916
Island	CO	106,826	971
Island	CT	105,206	885
Island	IR	105,164	920
Continent	GE	91,776	847

*N*_*e*_ were estimated as the nucleotide diversity π divided by four times the mutation rate, estimated as ${{\boldsymbol{4}}{\boldsymbol{.}}{\boldsymbol{6}}\,{{\times}}\,{\boldsymbol{10}}}^{{\boldsymbol{-}}{\boldsymbol{9}}}$ (Smeds et al. 2016). We performed 1000 bootstraps by sampling only unrelated individuals for all populations. Standard-error (SE) around the *N*_*e*_ mean indicates the variation among the different bootstraps.

### Probability of belonging to a HBD segment

**Fig. 3:**
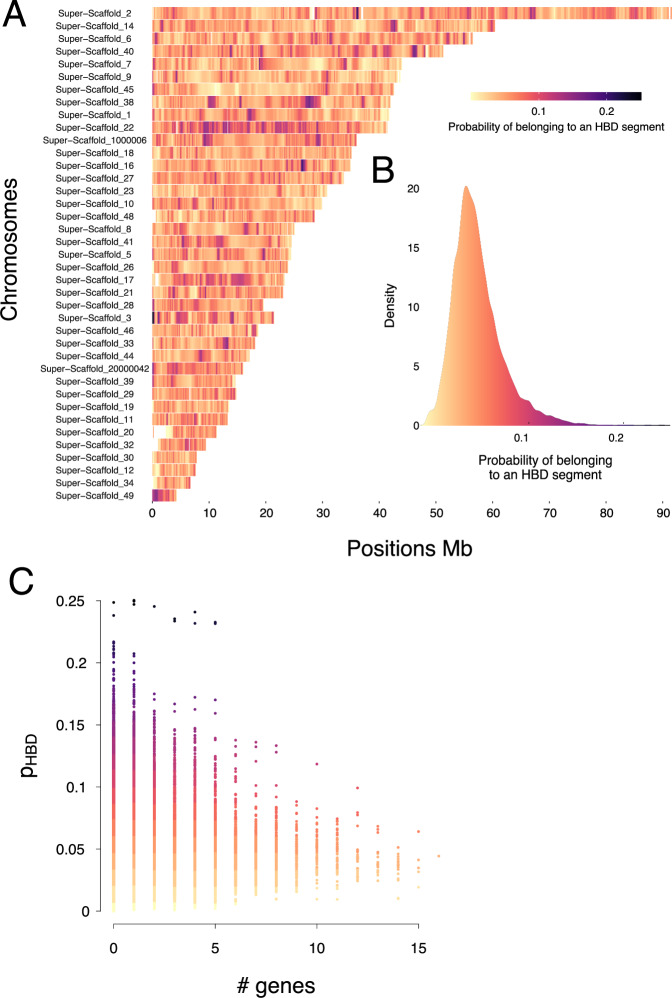
Quantification of the probability to belong to an HBD segment along the genome. **A** Probability to belong to an HBD segment coalescing less than 512 generations ago along the different super-scaffolds. Probabilities were estimated via overlapping 100 kb windows with a 20 kb sliding step. Blank spaces correspond to windows where no SNPs were present. **B** Density of probability to belong to an HBD segment, all super-scaffolds included. The code to create this figure was obtained from M. Stoffel GitHub. **C** Probability that a 100 Kb window is HBD according to the number of genes in this window.

To see whether specific genomic regions are enriched in deleterious alleles and whether these regions are randomly distributed along the genome, we quantified the occurrence of HBD segments along the genome. For the vast majority of the genome, the probability of belonging to a HBD segment is low (<0.1) (Fig. 3). However, a few super-scaffolds (such as super-scaffold 22) and specific genomic regions, most notably at the beginning of super-scaffold 3, show particularly high HBD probabilities. We investigated the genes present at the beginning of this super-scaffold 3 further, but did not identify any pattern. We show in panel C that gene rich windows are very unlikely to be autozygous. Finally, we identify regions with extremely high (HBD islands) and low (HBD deserts) HBD probabilities (Tables S3, S4). HBD islands and deserts were definedas windows in the higher or lower 2.5% of HBD probabilities respectivly. Gene ontology enrichment analyses in HBD segments islands and deserts reveal no specific enrichment in these regions.

### Accumulation of minor alleles

**Fig. 4:**
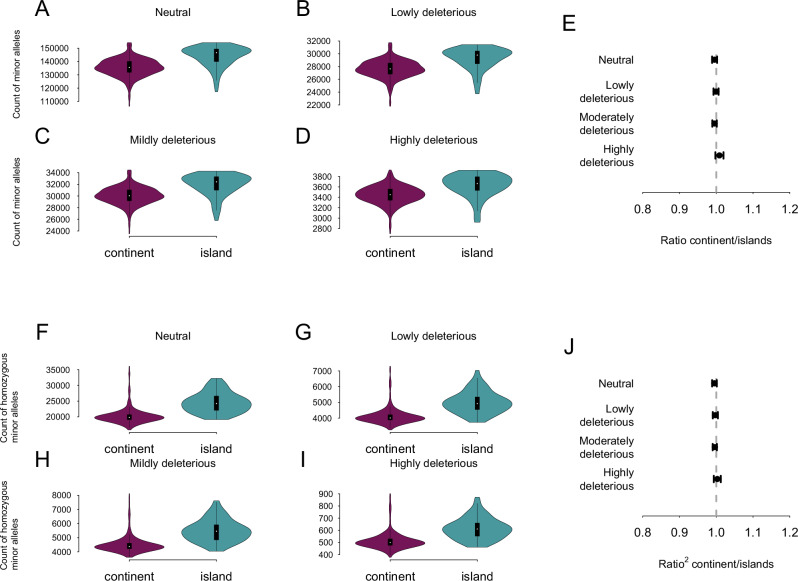
Distribution of minor alleles in continental versus islands populations according to their deleterious effects. Minor allele effects were classified with SnpEff. **A** Count of neutral minor alleles. **B** Count of lowly deleterious minor alleles. **C** Count of moderately deleterious minor alleles. **D** Count of highly deleterious minor. **E** ${R^{\prime} }_{{XY}}$ Ratio of minor alleles in continental populations to island populations scaled by the same ratio for SNPs located in intergenic regions. ${R^{\prime} }_{{XY}}$ < 1 indicates that insular populations are more enriched in minor alleles of the focal category compared to their enrichment for neutral intergenic alleles. On the contrary, ${R^{\prime} }_{{XY}}$ > 1 indicates that insular populations are depleted in minor alleles of the focal category compared to their enrichment for intergenic alleles. **F** Count of homozygous neutral minor alleles. **G** Count of homozygous lowly deleterious minor alleles. **H** Count of homozygous moderately deleterious minor alleles. **I** Count of homozygous highly deleterious minor alleles. **J** ${R^{\prime} }_{{XY}}^{2}$ ratio of minor alleles in continental populations compared to island populations. ${R^{\prime} }_{{XY}}^{2}$ < 1 indicates that insular populations are more enriched in homozygous minor alleles of the focal category compared to their enrichment for neutral homozygous intergenic alleles. On the contrary, ${R^{\prime} }_{{XY}}^{2}$ > 1 indicates that insular populations are depleted in homozygous minor alleles of the focal category compared to their enrichment for homozygous intergenic alleles. For violin plots, continental populations are shown in purple and island populations in blue.

To assess whether there is a difference in selection efficiency between islands and continental populations, we quantified the number of minor alleles (as proxy for deleterious alleles) in each individual (Fig. 4). Figure 4A–D shows the number of minor alleles per variant category (A: Neutral; B: Lowly deleterious, C: Moderately deleterious; D: Highly deleterious) in continental populations versus island populations. Island populations were significantly enriched in minor alleles for all variants categories (Wilcoxon rank sum tests; Neutral: W = 6537.5, *p*-value < 2.2e-16, effect size = 0.414, considered moderate; Lowly deleterious: W = 7022.5, *p*-value < 2.2e-16, effect size = 0.396, considered moderate; Moderately deleterious: W = 6907.5, *p*-value < 2.2e-16, effect size = 0.400, considered moderate; Highly deleterious: W = 8084, *p*-value = 1.189e-15, effect size = 0.357, considered moderate). This enrichment remained present when we controlled for individual genetic diversity (by dividing the count of minor alleles by the number of polymorphic sites per individual) (Fig. S8A–D). Interestingly, the lower tails of the insular violin plots consistently contained the individuals from GB and IR, suggesting that their minor allele counts are more similar to continental populations than to those from the other islands. Concerning the enrichment of each of these categories in respect to their enrichment in intergenic mutations, we consider a ratio significant if the standard errors around its estimation do not overlap with one. The ratios (continents/islands) were not significantly different from one for all mutation types (Fig. 4E). This shows that island populations are similarly enriched in all allele categories compared to intergenic regions. Figure 4F–I shows the number of homozygous minor alleles per variant category (F: Neutral; G: Lowly deleterious, H: Moderately deleterious; I: Highly deleterious) in continental populations versus island populations. Island populations were also significantly enriched in homozygous minor alleles for all variants categories (Wilcoxon rank sum tests; Neutral: W = 4034.5, *p*-value < 2.2e-16, effect size = 0.505, considered large; Lowly deleterious: W = 4482, *p*-value < 2.2e-16, effect size = 0.489, considered moderate; Moderately deleterious: W = 4314.5, *p*-value < 2.2e-16, effect size = 0.495, considered moderate; Highly deleterious: W = 5269.5, *p*-value < 2.2e-16, effect size = 0.460, considered moderate), even after correction for individual genetic diversity (Fig. S8E–H). The ratio (continent/islands) were not significantly different from one for all homozygous minor alleles.

**Fig. 5:**
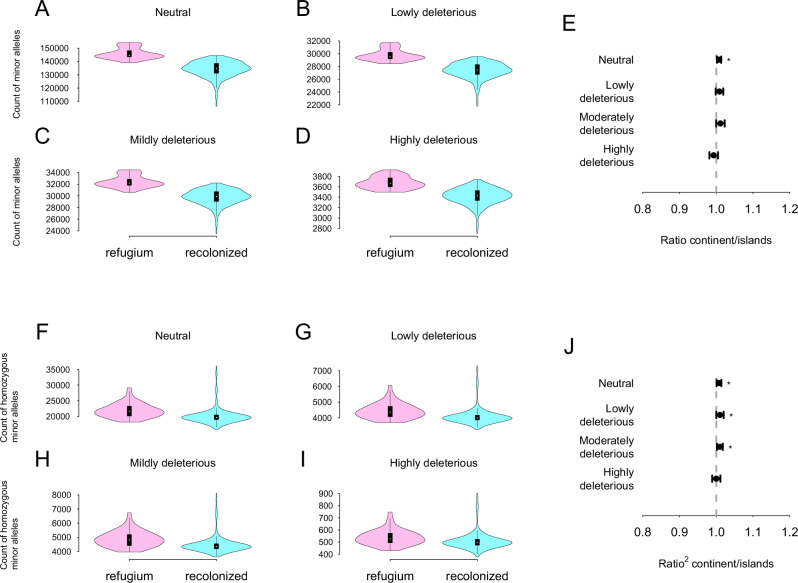
Distribution (among individuals) of minor alleles in continental refugium populations versus recolonized (after the last glacial maximum) populations. Minor allele effects were estimated with SnpEff. **A** Count of neutral minor alleles. **B** Count of lowly deleterious minor alleles. **C** Count of moderately deleterious minor alleles. **D** Count of highly deleterious minor alleles. **E** ${R^{\prime} }_{{XY}}$ Ratio of minor alleles in refugium populations compared to recolonized populations. ${R^{\prime} }_{{XY}}$ < 1 indicates that recolonized populations are enriched in minor alleles of the focal category compared to their enrichment for supposedly neutral intron alleles. On the contrary, ${R^{\prime} }_{{XY}}$ > 1 indicates that recolonized populations are more depleted in minor alleles of the focal category compared to their enrichment for intron alleles. **F** Count of homozygous neutral minor alleles. **G** Count of homozygous lowly deleterious minor alleles. **H** Count of homozygous moderately deleterious minor alleles. **I** Count of homozygous highly deleterious minor alleles. **J** ${R^{\prime} }_{{XY}}^{2}$ ratio of minor alleles in refugium populations compared to recolonized populations compared to the baseline enrichment for supposedly neutral intron alleles. ${R^{\prime} }_{{XY}}^{2}$ < 1 indicates that recolonized populations are enriched in homozygous minor alleles of the focal category compared to their enrichment in homozygous intron alleles. On the contrary, ${R^{\prime} }_{{XY}}^{2}$ > 1 indicates that recolonized populations are more depleted in homozygous minor alleles of the focal category compared to their enrichment in homozygous intron alleles. For violin plots, refugium populations are shown in pink and recolonized populations in light blue.

Similarly to Fig. 4, we compared the number of minor alleles (as proxy for deleterious alleles) in each individual and therefore the selection efficiency between continental refugia and recolonized populations (Fig. 5). Figure 5A–D shows the number of minor alleles per variants category (A: Neutral; B: Lowly deleterious, C: Moderately deleterious, D: Highly deleterious) in continental refugium populations (during the last glacial maxima) versus continental recolonized populations. Recolonized populations were significantly depleted in minor alleles for all variants categories (Wilcoxon rank sum tests; Neutral: W = 16,067, *p*-value < 2.2e-16, effect size = 0.512, considered large; Lowly deleterious: W = 15,998, *p*-value < 2.2e-16, effect size = 0.508, considered large; Moderately deleterious: W = 15,997, *p*-value < 2.2e-16, effect size = 0.508, considered large; Highly deleterious: W = 15,151, *p*-value < 2.2e-16, effect size = 0.453, considered large). However, this is solely due to the higher genetic diversity of refugium populations and this depletion disappeared when we corrected for individual genetic diversity (by dividing the count of minor alleles by the individual number of polymorphic sites) (Fig. S9A–D). Concerning the ${R^{\prime} }_{{XY}}$ ratios (refugium/recolonized), it was significantly higher than one for neutral minor alleles, possibly reflecting the lack of neutral genetic diversity in recolonized populations. On the contrary, ${R^{\prime} }_{{XY}}$ ratios were not significantly different from one for all types of deleterious minor alleles (Fig. 5E), indicating that recolonized continental populations are as enriched in deleterious minor alleles compared to intergenic minor variants. Figure 5F–I shows the number of homozygous minor alleles per variant category (F: Neutral; G: Lowly deleterious, H: Moderately deleterious; I: Highly deleterious) in refugium populations versus recolonized populations. Recolonized populations were also significantly enriched in homozygous minor alleles for all variants categories (Wilcoxon rank sum tests; Neutral: W = 12,796, *p*-value = 9.64e-10, effect size = 0.300, considered small; Lowly deleterious: W = 12,364, *p*-value = 3.005e-08, effect size = 0.272, considered small; Moderately deleterious: W = 12,540, *p*-value = 7.688e-09, effect size = 0.283, considered small; Highly deleterious: W = 11,056, *p*-value = 0.0001399, effect size = 0.187, considered small). Similarly to what we observed for the count of minor alleles, this depletion disappeared when we divided the number of homozygous minor alleles by the individual genetic diversity for all categories of alleles (Fig. S9E–H). Concerning the ${R^{\prime} }_{{XY}}^{2}$ ratios (refugium/recolonized), recolonized populations were less enriched than refugium populations in minor homozygous alleles for neutral as well as lowly and mildly deleterious variants but the ratio was not significantly different from one for highly deleterious variants (Fig. 5J).

## Discussion

In this study, we examine the status and origin of inbreeding in barn owls across Europe and compare continental and island populations. We make use of homozygous-by-descent (HBD) segments which are proxy for identical-by-descent (IBD) segments identified with a model-based approach (see introduction). Our findings indicate that insular populations have higher *F*_*HBD*_ compared to continental populations and the inbreeding of island individuals is largely a result of ancient coalescence events (probably due to both founders effects and lower effective population sizes). Since, *F*_*HBD*_ is higher than *F*_*AS*_ this indicates that the populations are mostly mating randomly but are still enriched for identical-by-descent (IBD) segments. Additionally, homozygous-by-descent (HBD) segment distributions demonstrate that island populations exhibit higher numbers of small fragments indicating more ancient coalescence events (Thompson 2013; Speed and Balding 2015). These results are consistent with the rank of the estimated *N*_e_, as well as with a study which showed that the population from CT showed higher inbreeding compared to CY, AE, GR and IS, was enriched in small ROHs, and mated mostly randomly (*F*_*IS*_ = -0.018) (Machado, Topaloudis, et al. 2022). A previous study found that both populations from the Canary islands are less heterozygous than PT and MA and mostly mated randomly (*F*_*IS*_ = −0.015 and −0.030) (Cumer et al. 2022, 2022) which further supports our findings here. Typically, insular populations are more inbred due to their smaller size, isolation, and colonization often occurs from a small number of individuals - also called founder effect (Keller and Waller 2002). Interestingly, the GB and IR populations had more HBD segments for the same fraction of genome being autozygous compared to other island populations. This indicates that even though the fraction of genome within HBD segments is the same, their fragments are on average smaller and closer to the HBD segments distributions from continental populations. In addition, the number of minor allele counts was more similar to the values estimated from continental populations than values estimated from other island populations. The colonization of GB anf IR is fairly recent compared to the other islands (occurring after the last glacial maxima) and they also exhibit a homogeneous genetic structure, all of which is consistent with a history of small *N*_e_ (Machado, Cumer, et al. 2022).

The majority of individuals from continental populations were sampled in Switzerland (CH). This population is part of a long term study which records phenotypic and pedigree information for individuals within a study area (Roulin et al. 1998; San-Jose et al. 2019; Béziers and Roulin 2021). Sampling focusses on families and individuals frequently return or remain in the study area resulting in a family structure within the records. This structure was then further confirmed by comparing *F*_*AS*_ and *F*_*HBD*_: most CH individuals were below the identity line (Fig. 2). Additionally, we identified 14 highly inbred individuals that were the result of mating events between closely related parents in accordance with the pedigree.

The French (FR) population showed a higher mean *F*_*HBD*_ and an enrichment in the HBD segment class due to recent coalescence events, and a lower *N*_e_ estimate. Inbreeding and *N*_e_ estimates were likely biased due to one individual (out of five) that was strongly inbred, resulting in a high mean *F*_*HBD*_ and recent HBD segment sum of lengths. We observed unexpected results for the population from Georgia (GE) which displayed very low *N*_e_ and negative *F*_*AS*_. This is most likely due to these samples being full-siblings and therefore sharing more alleles than unrelated individuals, which will influence the estimation of *π* and *N*_e_. As a result, their *F*_*AS*_ is also affected: the sampled population average allele matching is exceptionally high (even after relatedness filtering), but because their parents are not closely related, they do not share many more homozygous sites than expected under random mating, which causes a low allele matching score multiplied by a very high relatedness average which leads to a negative *F*_*AS*_. Consequently, with the current samples, we cannot draw any conclusions about what the effective population size estimate as well as the origin of inbreeding in this French population. However, the remaining analyses such as *F*_*HBD*_ estimation as well as the count of minor alleles should not be biased. Compared to more northern continental populations, Portugal (PT), Morocco (MA) and Israel (IS) had higher *N*_e_ estimates and lower HBD segments sum of lengths. This is because these populations are the largest refugia (Cumer et al. 2021). Although Italy (IT) was also identified as a refugium, its size is smaller, which may explain the smaller *N*_e_ and enrichment in small HBD segments. In addition, the sampling in Italy was quite localized and not representative of the whole country. There was little difference in inbreeding levels between recolonized continental populations and refugium continental populations, which is likely due to constant and strong gene flow between both groups, as shown by the low *F*_*ST*_ (0.047) among populations (Cumer et al. 2021).

While the absolute *N*_e_ values we estimated cannot be used to draw any conclusions as they are unusually high and thus likely unrealisitc, relative comparisons between our populations should still be valid.The high estimated *N*_e_ values may be due to a number of different factors. Firstly, we have strong connectivity among all our populations (the maximum *F*_*ST*_ is between CY and PT and is equal to 0.102 (Cumer et al. 2021)). It is therefore likely that neighboring populations are exchanging large numbers of migrants per generation. It has been shown that strong gene flow can inflate population-specific *N*_e_ estimation to the metapopulation *N*_e_ value (Waples and England 2011). Furthermore, the AE’s very high *N*_e_ estimate can also be attributed to the fact that this population barely differs from the large continental GR population (*F*_*ST*_ = 0.014 (Machado, Topaloudis, et al. 2022)). In addition, CY showed a particularly high *N*_e_ compared to other islands. This is in agreement with previous studies, who determined that CY is the most diverse island in the Mediterranean Sea and has the strongest gene flow with the mainland (Machado, Topaloudis, et al. 2022). Finally, the estimation of N_e_ is based in part on the mutation rate, although we did not have an estimation of the mutation rate for barn owls. Therefore, we used an estimation from the collared flycatcher (*Ficedula albicollis*) from only one family (Smeds et al. 2016), which may have caused our result to be upward biased.

The probability (averaged among individuals) that a genomic region is HBD varied along the genome but was generally quite low, indicating low average inbreeding levels in the wild barn owls. This is especially true when compared to another species where the same probability was estimated: a Soay sheep population (Stoffel et al. 2021). It is anticipated, however, that barn owl populations are less inbred than the Soay sheep which only connsisted in a small isolated population with high relatedness (Stoffel et al. 2021). We further showed that, in the barn owl, the probability of a region being HBD was strongly and negatively correlated with the number of genes in the region: regions with high gene density had a lower probability of being HBD. Therefore, there is a constraint on regions with high gene density to have lower homozygosity probabilities. This is in agreement with inbreeding depression theory, which predicts that homozygosity at a coding site will result in the expression of recessive deleterious alleles, thereby reducing the fitness of individuals (Charlesworth and Willis 2009). As a result, selection will act to reduce homozygosity in these regions if they contain recessive deleterious alleles. There is no evidence that the high probability of belonging to an HBD segment at the beginning of Super-Scaffold 3 is biologically significant. The high probability of being HBD could be an artifact of the higher coverage of this region, probably itself an artifact of genomic region duplication. Indeed regional coverage was twice as much as the rest of the genome (in both our data and the genome assembly. Duplication increases the chance of finding homozygosity, which in turn, increases the probability that a region is HBD.

We showed that most inbreeding is due to small effective population sizes rather than mating between closely related individuals. According to Glémin (2003), purging solely due to a reduction in population size has been shown to occur only when population size is small enough for drift to increase homozygosity and cause recessive deleterious alleles to be expressed in the majority of individuals (Charlesworth and Willis 2009), without being too small to prevent drift from overcoming selection.

Our findings demonstrate that insular populations were enriched in all types of minor alleles in terms of absolute numbers, likely as a result of drift, suggesting that selection has been less efficient at removing deleterious alleles in island populations. In addition, insular populations were similarly enriched for all types of intragenic variants compared to intergenic mutations. This indicates that selection has also been less efficient at removing deleterious alleles in the smaller insular populations for all types of deleterious mutations. It is interesting to note that even though it is not significantly different from one, the average ratio of continental and island populations for highly deleterious mutations is slightly higher than one although, our estimates are still far from the values which have been reported as evidence for purging in the wild in mountain gorillas as (${R}_{{XY}}^{{\prime} }$ = 0.8) (Xue et al. 2015) and Alpine ibex (${R}_{{XY}}^{{\prime} }$ = 0.525) (Grossen et al. 2020). We should, however, note that the bottlenecks of these species were extremely severe compared to what occurred during the colonization of islands by barn owls: the mountain gorilla population size was estimated at around 800 individuals in 2015 (Xue et al. 2015) while the Ibex Swiss population has been reintroduced from only 100 individuals with little subsequent gene flow (Grossen et al. 2020). We therefore hypothesize that the island populations that are the focus of our study are too large to facilitate purging of deleterious mutations. The same type of enrichment across all categories of deleterious alleles was also observed in the human out-of-Africa expansion (Peischl et al. 2013; McCoy and Akey 2016) but, similarly to what we report in this study, no evidence of purging of highly deleterious alleles was detected (Do et al. 2015).

Interestingly, all categories of deleterious minor alleles were depleted in recolonized continental populations compared to refugium populations. However, this depletion was not consistent when we considered individual genetic diversity which is known to be higher in populations inhabiting the refugia (Cumer et al. 2021). Recolonized populations were significantly less depleted in neutral minor alleles compared to intergenic minor alleles (but the ratio was very close to 1) likely reflecting their lower genetic diversity. This pattern was, however, not observed for all types of deleterious mutations, which suggests that selection has been as efficient at removing deleterious alleles for all categories. Overall, the depletion of minor alleles in recolonized populations is in contradition with what has been reported in the literature (Peischl et al. 2013; Henn et al. 2016; Rougemont et al. 2023). This could be explained because the continental populations were recolonized long ago and are not at the extremities of range expansion anymore. In addition, there were multiple source populations for Europe mainland recolonization (Cumer et al. 2021) which could reduce the expansion load (Peischl et al. 2013). Furthermore, recolonized continental populations have constant gene flow with the source populations (here the refugium), thus reducing the possibility that rare alleles will rise to high frequencies (Nigenda-Morales et al. 2023). Finally, we want to highlight that the different SnpEff categories we used have the potential to be biased. Indeed, it is very hard to merge different types of mutations into lowly, mildly or highly deleterious variants and be confident about their effect. Some authors have preferred to use more precise categories of variants such as loss of functions variants (as proxy for highly deleterious mutations), synonymous mutations (as proxy for neutral or lowly deleterious variants) or non-synonymous mutations (as proxy for mildly deleterious variants) (Humble et al. 2023; Nigenda-Morales et al. 2023).

## Conclusion

In this study, we examined the inbreeding status and the HBD segments landscape of barn owl populations throughout Europe. Compared to continental populations, insular populations are more inbred, and the inbreeding primarily results from a small effective population size rather than recent consanguinity. We show that the probability that a region is autozygous diminishes with the number of genes present in this region. Finally, we show that in comparison to continental populations, island populations are enriched in all deleterious categories of minor alleles, reflecting the lower efficiency of selection at removing deleterious alleles in smaller populations and an absence of efficient purging. If certain island populations are indeed too big for efficient purging of deleterious alleles, this raises an important consideration for conservation. Only small populations with an effective population size of a few hundred or less can undergo purging, therefore, conservation strategies for species that are not categorised as critically endangered are unlikely to benefit from the efficient purging that is found in these smaller populations. Moreover, such small populations undergoing purging of highly deleterious alleles will usually also incur an additional cost of increased lowly and mildly deleterious mutations. Nevertheless, it does appear that in the absence of purging, whole-genome homozygosity correlates positively with deleterious homozygosity and can therefore serve as a reliable proxy for a population genetic load.

## Supplementary information

Supplementary Material file

Table S1

Table S2

Table S3

Table S4

## Data Availability

The data used in this study have been previously published in Cumer et al. (2021, 2022, 2022, 2024) and Machado, Cumer, et al. (2022) and are available in https://www.ncbi.nlm.nih.gov/sra, BioProjects PRJNA694553, PRJNA700797, PRJNA727977, PRJNA727915, PRJNA774943 and PRJNA925445. Data for the populations from Georgia and Corsica have been originally used and described in (Topaloudis et al. 2024) submitted recently and will be available with their own BioProject soon.
