# Supplementary material for: Too big to purge: persistence of deleterious Mutations in Island populations of the European Barn Owl (Tyto alba)
Source: Heredity (Edinb). 2024 Oct 13;133(6):437–49. doi: 10.1038/s41437-024-00728-8 (PMC11589586; doi:10.1038/s41437-024-00728-8)
Supplement: Supplementary file 1 — Supplementary Material file [file 41437_2024_728_MOESM1_ESM.docx]

**Supplementary Material: Too big to purge: persistence of deleterious mutations in island populations of the European barn owl (*Tyto alba*).**

**Authors:** Eléonore Lavanchy, Tristan Cumer, Alexandros Topaloudis, Anne-Lyse Ducrest, Céline Simon, Alexandre Roulin and Jérôme Goudet

Supplementary Material and Methods

**Trimming relatedness**

The 502 individuals were trimmed for allele-sharing based relatedness *>* 0.05 with a custom R script and the hierfstat R package (Goudet, 2005). 187 individuals were left after relatedness trimming.

**Estimating additional inbreeding coefficients**

*F_PED_* was estimated as twice the diagonal of the kinship matrix minus 1. The kinship matrix was estimated with the *kinship* function from the kinship2 R package.

**Comparing minor and derived allele identification**

As mentioned in the main text, we compare the fraction of sites for which the minor allele (we identified by bootstrapping individuals) corresponds to the derived allele in a set of 1,373,932 neutral sites from (Machado, Cumer, *et al.*, 2022). We show that the mean fraction of correct assignment is 87% but that this fraction decreases linearly with site’s MAF (Figure S1).

**Comparing *F_AS_* and *F_HBD_***

As mentioned in the main text, we use the comparison between population specific *F_AS_* and *F_HBD_* to identify the original time of inbreeding. This method is an extension of the approach used by Clark *et al.*, (2019) in their supplementary Figure S4. There, they compared the mean FROH of a population to its *F_IS_* to draw similar conclusions. We propose to use population specific individuals’ *F_AS_* rather than the population *F_IS_* because they have similar properties, but *F_AS_* allow to retain the variability among the individuals of a population and thus contain more information.

**Figure S1:** Fraction of minor allele which correspond to the derived allele. Minor allele was estimated globally via 1,000 bootstraps by sampling only unrelated individuals. The size of the dots corresponds to the number of alleles in each MAF category.

Supplementary Results

**Figure S2:** Cumulative *F_HBD_* (estimated as the average (among variants) prob- ability that a variant belongs to an HBD segment coalescing less then 512 generations ago) distributions among the different HBD classes used in RZooRoH. Continental populations are colored in purple and island populations in blue.

This plot shows the cumulative *F_HBD_* across the different HBD classes used in the RZooRoH model (Druet and Gautier, 2017, 2022; Bertrand *et al.*, 2019). We show that HBD segments coalescing less than 8 generations ago are very rare and mostly present in the truly inbred individuals mentioned above. Difference between continental and islands populations start to show from HBD segments coalescing around 16 generations ago. Islands *F_HBD_* especially increase when coalescence events from 64 generations are included. In the main text analyses, we only consider SNPs or segments HBD when they are included in the first 10 classes (from 1 generation ago to 512 generations ago).

**Figure S3:** *F_HBD_* (estimated as the average (among variants) probability that a variant belongs to an HBD segment coalescing less then 512 generations ago) distributions per population. Continental populations are colored in purple and island populations in blue.

On average, islands populations show higher *F_HBD_* compared to continental populations. We detect few truly inbred individuals (resulting from mating between close relatives): 16 from CH, 1 from PT, one from FR, one from GB and one from EC. Among continental populations, individuals from GE, SB and GR shows highest *F_HBD_*. Among island populations, individuals from AE and CY showed the lowest *F_HBD_*.

**Figure S4:** For all panels, refugia populations are shown in pink and recolonized populations in blue. **A:** *F_HBD_* distributions from refugia and recolonized populations. *F_HBD_* considers a marker as autozygous if the coalescence event is before 512 generations ago. **B**: scatter plot of *F_HBD_* against *F_AS_*. Each point represents one individual and its shape indicates which population it comes from. The black line is the identify line (x = y). **C**: number of HBD segments (*N_HBD_*) as a function of the mean length of HBD segments (*S_HBD_*) in base-pair. Each point represents one individual and its shape indicates which population it comes from. **D**: HBD segments distributions from refugia populations and recolonized populations. The y-axis represents the mean sum of length (among individuals) falling into the different categories of HBD segments (represented in the x-axis).

There is little (but significant) difference between continental refugium and recolonized populations *F_HBD_* distribution (Wilcoxon rank-sum test: W = 10’063, p-value = 0.01275; effect size: 0.122, considered small). For instance, there is no strongly inbred individuals in refugium populations. In addition, both refugium and recolonized populations are equally close to the *F_HBD_* and *F_AS_* identity line (except the Swiss individuals which are largely below). Refugium populations tended to have smaller HBD fragments for the same fraction of genome within HBD segments compared to recolonized populations (except for one inbred Greek individual). Finally, the refugium populations were slightly enriched in small HBD segments (coalescing 128 and 256 generations ago). On the contrary recolonized populations were enriched in both large (coalescing 8 generations ago) and very small (coalescing 512 generations ago) HBD segments but the enrichment in large HBD segments is solely due to the few inbred Swiss present in the recolonized populations.

**Figure S5:** *F_HBD_* according to *F_AS_* in the set of 187 unrelated individuals. Each point represents one individual and its shapes indicates the population it comes from. The black line represents the identify line (x = y). Continental populations are colored in purple and island populations in blue.

This plot compares *F_HBD_* and *F_AS_* inbreeding coefficients with the unrelated set of individuals. Most individuals below the line have been removed or shifted towards the line, however few individuals are still below the line. We believe it is due to that fact that we did not filter on relatedness 0 (bur rather 0.05). In addition, the Swiss sample we have is much larger compared to other populations and is known to contain families. We also know that MA samples are somehow related.

**Figure S6:** Comparison between *F_HBD_* (estimated as the average (among variants) probability that a variant belongs to an HBD segment coalescing less then 512 generations ago) and *F_PED_* in the Swiss population (CH).

This plot shows the comparison between the genetic observed *F_HBD_* and the expected *F_PED_* for the CH population. We show that there is a good correlation between both inbreeding coefficients (0.94).

**Figure S7:** HBD segments distributions per population. Populations order is as follow: CH, DK, FR, IS, PT, IT, MA, GE, SB, GR, AE, IO, CO, CT, CY, EC, WC, GB, IR. The y axis represents the mean sum of length (among individuals) falling into the different categories of HBD segments (represented in the x axis).

We show HBD segments distributions among the different populations. In populations with small sample sizes, the peaks of mean sum of lengths are mostly driven by inbred individuals (namely in FR for HBD classes 5 and 6 and EC for HBD class 6 and 7). We observe that CT population has an especially high peak in the 8th HBD class coherent with its history of isolation and small effective population size.

**Figure S8:** Distribution of minor alleles in continental versus islands populations. Minor alleles effects were classified with SnpEff. **A:** Count of neutral minor alleles divided by the individual number of polymorphic sites. **B:** Count of lowly deleterious minor alleles divided by the individual number of polymorphic sites. **C:** Count of moderately deleterious minor alleles divided by the individual number of polymorphic sites. **D:** Count of highly deleterious minor alleles divided by the individual number of polymorphic sites. **E:** Count of homozygous neutral minor alleles divided by the individual number of polymorphic sites. **F:** Count of homozygous lowly deleterious minor alleles divided by the individual number of polymorphic sites. **G:** Count of homozygous moderately deleterious minor alleles divided by the individual number of polymorphic sites. **H:** Count of homozygous highly deleterious minor alleles divided by the individual number of polymorphic sites.

This plot shows the individual count of minor alleles divided by the individual number of polymorphic sites per variants category (A: Neutral; B: Lowly deleterious, C: Moderately deleterious; D: Highly deleterious; E: homozygous neutral; F: Homozygous lowly deleterious, G: Homozygous moderately deleterious; H: Homozygous highly deleterious) in continental populations versus island populations. Islands populations are significantly enriched in all types of minor alleles both for single copy and homozygous states (Neutral single copy: W = 6904, p-value *<* 2.2e-16 Lowly deleterious single copy: W = W = 7739, p-value *<* 2.2e-16; Mildly deleterious single copy: W = 7410, p-value *<* 2.2e-16; Highly deleterious single copy: W = 9167, p-value = 1.07e-12; Neutral homozygous: W = 6752, p-value *<* 2.2e-16; Lowly deleterious single copy: W = 6748, p-value *<* 2.2e-16; Mildly deleterious single copy: W = 6613, p-value *<* 2.2e-16; Highly deleterious single copy: W = 7555, p-value *<* 2.2e-16).

**Figure S9:** Distribution of minor alleles in refugium versus recolonized populations. Minor alleles effects were classified with SnpEff. **A:** Count of neutral minor alleles divided by the individual number of polymorphic sites. **B:** Count of lowly deleterious minor alleles divided by the individual number of polymorphic sites. **C:** Count of moderately deleterious minor alleles divided by the individual number of polymorphic sites. **D:** Count of highly deleterious minor alleles divided by the individual number of polymorphic sites. **E:** Count of homozygous neutral minor alleles divided by the individual number of polymorphic sites. **F:** Count of homozygous lowly deleterious minor alleles divided by the individual number of polymorphic sites. **G:** Count of homozygous moderately deleterious minor alleles divided by the individual number of polymorphic sites. **H:** Count of homozygous highly deleterious minor alleles divided by the individual number of polymorphic sites.

This plot shows the individual count of minor alleles divided by the individual number of polymorphic sites per variants category (A: Neutral; B: Lowly deleterious, C: Moderately deleterious; D: Highly deleterious; E: homozygous neutral; F: Homozygous lowly deleterious, G: Homozygous moderately deleterious; H: Homozygous highly deleterious) in refugium populations versus recolonized populations. There is no significant difference between both groups of populations for all types of minor alleles both for single copy and homozygous states (Neutral single copy: W = 8125, p-value = 0.9382; Lowly deleterious single copy: W = 8161, p-value = 0.9762; Mildly deleterious single copy: W = 8383, p-value = 0.7924; Highly deleterious single copy: W = 6857, p-value = 0.0786; Neutral homozygous: W = 8518, p-value = 0.6583; Lowly deleterious single copy: W = 8334, p-value = 0.8429; Mildly deleterious single copy: W = 8795, p-value = 0.4182; Highly deleterious single copy: W = 7331, p-value = 0.2583).

**Comparing populations in details**

When we zoom into the population-specific *F_HBD_* (Figure S2), mean sum of HBD lengths per HBD class (Figure S6), and *N*_e_ estimation (Table 1), the pattern differs among populations. Concerning islands populations, both the EC and WC populations displayed high *F_HBD_* (mean *F_HBD_* = 0.132 and 0.112 for EC and WC respectively, with large variance among individuals for the EC though), were enriched in HBD classes from 32g to 128g ago. Additionally, their *N*_e_ estimation was higher than the other islands. We found that the AE population had low HBD coefficients (mean *F_HBD_* = 0.061), very similar to those from GR, was slightly enriched in HBD segments coalescing 32g and 64g ago, and had very high *N*_e_ estimation, which were very similar to GR. The distribution of HBD segments in the IO population was very similar to that of the AE populations, but they had slightly longer sums of lengths for segments coalescing 256g ago. Additionally, individuals from the IO population displayed slightly higher *F_HBD_* values (mean *F_HBD_* = 0.081) and lower *N*_e_ estimates. In the CO population, *F_HBD_* coefficients were high (mean *F_HBD_* = 0.098) and the HDB segments distribution was highly enriched in segments coalescing 256 generations ago. In addition, the *N*_e_ estimation for the CO population was among the lowest. From all the populations studied, CT appeared to be one of the most inbred with low *N*_e_, high *F_HBD_* values (mean *F_HBD_* = 0.134) and a substantial percentage of HBD segments from 128 generations ago. Compared to other islands, the CY population had lowest *F*_HBD_ coefficients (mean *F_HBD_* = 0.073) and was enriched in HBD segments from 64g and 128g ago. In addition, the CY *N*_e_ estimate was relatively high compared to those of the other islands. Finally, the IR and GB populations had high *F*_HBD_ (mean *F_HBD_* = 0.104 and 0.102 for GB and IR, respectively), the lowest *N*_e_ estimation, and were enriched for HBD segments dating back to 256g and 512g.

Concerning continental populations, they displayed lower FHBD and higher Ne estimation. CH and DK individuals displayed low inbreeding coefficient. Concerning continental populations, they displayed lower *F_HBD_* and higher *N*_e_ estimation. CH and DK individuals displayed low inbreeding coefficients (mean *F_HBD_* = 0.040 and 0.041 respectively), high *N*_e_ estimations, and were highly enriched in HBD segments that coalesced over 512 million years ago. In contrast to the other continental populations, the FR population had high values of *F_HBD_* (mean *F_HBD_* = 0.092) and was enriched in HBD segments that coalesced 16g, 32g, 256g and 512g ago. It showed, however, high *N*_e_ estimates comparable to those of the rest of the continental populations. In the IS, PT, and MA populations, there were low *F_HBD_* values (mean *F_HBD_* values are 0.042, 0.024, and 0.011 for IS, PT, and MA, respectively), no enrichment in any HBD segment class, and a high estimated *N*_e_ value. The mean *F_HBD_* of the GE population was the highest among continental populations (mean *F_HBD_* = 0.069). Furthermore, compared with other continental populations, their sum of HBD segment lengths was also enriched for segments that coalesced between 64g and 128g ago (although they were still smaller than most island populations). In addition, they displayed an extremely low estimated *N*_e_. The IT and SB populations had a mean *F_HBD_* of 0.046 and 0.054, respectively, and did not show any enrichment in any HBD segment classes. Similarly, their *N*_e_ estimation was very similar to the other recolonized continental populations. Finally, the GR population showed an average inbreeding coefficient value and a mean sum of HBD segment lengths (mean *F_HBD_* = 0.060). In addition, the GR *N*_e_ estimate was relatively large compared with other recolonized continental populations.

The AE displayed the highest *N*_e_ estimation, very close to the one from GR. This is concordant with previous studies which showed that there is low *F_ST_* between these two populations (Cumer *et al.*, 2021; Machado, Topaloudis, *et al.*, 2022). Concerning the IO population, the *N*_e_ estimation was slightly lower compared to the AE population (but higher than GB, IR, CO and CT) and HBD segments numbers and lengths were very similar to other island populations. Historically this population has been shown to be genetically close to GR as well (Machado, Topaloudis, *et al.*, 2022). The CY population also showed high *N*_e_ estimation and low *F_HBD_* distribution (compared to other islands). In addition, the number of HBD segments were the lowest among the island populations. The high diversity we found is consistent with previous studies and might be explained because this population is genetically close to the IS population (Cumer *et al.*, 2021; Machado, Topaloudis, *et al.*, 2022).The populations from CT and CO displayed even lower *N*_e_ and higher numbers of HBD segments. CT especially showed inflated *F_HBD_* distribution. This is consistent with its small size and stronger isolation from the rest of the populations (Machado, Topaloudis, *et al.*, 2022). On the contrary, CO showed especially high number of small HBD segments suggesting no recent mating between closely related individuals but an history of small *N*_e_ and long-term isolation. The Canary islands populations (EC and WC) showed *N*_e_ estimation close other small islands namely CT and CO and relatively high *F_HBD_* estimation, especially with one inbred individual in the EC population. Similarly, their sum of HBD segments lengths were clustering with the other island populations. All these are consistent with their history of old colonisation and long-term isolation (Cumer *et al.*, 2022). Populations from DK, IT and SB displayed *N*_e_, *F_HBD_* estimation and total numbers of HBD segments close to CH. This is not surprising as we know that there is shallow population differentiation between these populations (especially between CH and DK) (Cumer *et al.*, 2021). The population from GR displayed similar statistics compared to the rest of central Europe (namely CH, FR, DK, IT and SB) and had very similar estimates to the AE population. Concerning the MA population, we estimated a high *N*_e_ and very low inbreeding coefficients. This is concordant with previous studies which found that this population is close to the PT population (Cumer *et al.*, 2022) known as the biggest population and a refugium during the last glacial maxima (Cumer *et al.*, 2021). This is consistent with our results as it harbored the highest *N*_e_ and low *F_HBD_* in our analyses. In the MA population, we observed three individuals below the line in the *F_AS_* vs *F_HBD_* plot. These individuals are from the same family. Similar to what has been observed for the CH individuals, these individuals are shifted towards the line when we trim the data set for relatedness. Concerning the population from IS, we estimated a high *N*_e_ and a *F_HBD_* distribution as well as total number of HBD segments similar to what was observed for the CH, FR and DK populations. The high *N*_e_ is concordant with previous studies who found that the IS population is a reservoir of diversity (Cumer *et al.*, 2021; Machado, Topaloudis, *et al.*, 2022).

References

Bertrand AR, Kadri NK, Flori L, Gautier M, Druet T (2019). RZooRoH: An R package to characterize individual genomic autozygosity and identify homozygous-by-descent segments. *Methods in Ecology and Evolution* **10**: 860–866.

Clark DW, Okada Y, Moore KHS, Mason D, Pirastu N, Gandin I, *et al.* (2019). Associations of autozygosity with a broad range of human phenotypes. *Nature Communications* **10**: 4957.

Cumer T, Machado AP, Dumont G, Bontzorlos V, Ceccherelli R, Charter M, *et al.* (2021). Landscape and Climatic Variations Shaped Secondary Contacts amid Barn Owls of the Western Palearctic. *Molecular Biology and Evolution*: msab343.

Cumer T, Machado AP, Siverio F, Cherkaoui SI, Roque I, Lourenço R, *et al.* (2022). Genomic basis of insularity and ecological divergence in barn owls (Tyto alba) of the Canary Islands. *Heredity* **129**: 281–294.

Druet T, Gautier M (2017). A model-based approach to characterize individual inbreeding at both global and local genomic scales. *Mol Ecol* **26**: 5820–5841.

Druet T, Gautier M (2022). A hidden Markov model to estimate homozygous-by-descent probabilities associated with nested layers of ancestors. *Theoretical Population Biology* **145**: 38–51.

Goudet J (2005). Hierfstat, a Package for r to Compute and Test Hierarchical F-statistics. *Molecular Ecology Notes* **5**: 184–186.

Machado AP, Cumer T, Iseli C, Beaudoing E, Ducrest A-L, Dupasquier M, *et al.* (2022). Unexpected Post-Glacial Colonisation Route Explains the White Colour of Barn Owls (Tyto Alba) from the British Isles. *Molecular Ecology* **31**: 482–497.

Machado AP, Topaloudis A, Cumer T, Lavanchy E, Bontzorlos V, Ceccherelli R, *et al.* (2022). Genomic consequences of colonisation, migration and genetic drift in barn owl insular populations of the eastern Mediterranean. *Molecular Ecology* **31**: 1375–1388.
